# Breaking Down the Risk During Intravascular Lithotripsy: Is It the Lesion, the Lithotripsy, or the Lack of Intravascular Imaging?

**DOI:** 10.1016/j.jscai.2025.103799

**Published:** 2025-07-23

**Authors:** Arsalan Abu-Much, Ajay J. Kirtane

**Affiliations:** a Department of Medicine, Division of Cardiology, NewYork-Presbyterian Hospital/Columbia University Irving Medical Center, New York, New York; b Cardiovascular Research Foundation, New York, New York

**Keywords:** coronary artery calcification, intravascular imaging, intravascular lithotripsy

Coronary artery calcification continues to pose a significant challenge in percutaneous coronary intervention (PCI), as calcified lesions are technically more difficult to treat. PCI of calcified coronary lesions carries higher procedural risks such as dissection, perforation, and equipment noncrossing/entrapment and is associated with worse long-term outcomes, in part due to stent underexpansion. Also, there is a significant learning curve to the use of advanced calcium modification devices such as rotational and orbital atherectomy.

In part devised to address the limitations of conventional balloon angioplasty as well as to address the reticence to use more complex devices,^1^ intravascular lithotripsy (IVL) has emerged as a novel technique for modifying calcium using a balloon-based delivery system. By inducing both overt fractures and microfractures within calcified vessels through the application of acoustic energy generated using an electrical energy source, IVL increases lesion compliance in calcified lesions. This, in turn, allows for greater luminal gain and stent expansion, which should translate into improved long-term outcomes.^2^^,^^3^

While early studies demonstrated promising safety and efficacy for IVL, these studies were conducted in selected populations.^4^ The demonstrated efficacy of IVL in conjunction with its relative ease-of-use has led to rapid uptake of this device since its approval,^5^ yet real-world data on clinical outcomes associated with IVL, particularly in unselected and more complex patients and lesions, remain limited.

In this issue of *JSCAI*, van Oort et al^6^ present data from the BENELUX-IVL registry, a multicenter, all-comers cohort reporting on both short- and long-term outcomes in patients treated with IVL in real-world practice. The study includes over 500 patients treated across 8 European centers between 2019 and 2024, including approximately 30% of patients undergoing PCI for in-stent restenosis. While this heterogeneity enhances generalizability, lesions involving in-stent restenosis generally carry a lower risk of complications such as dissection when compared with de novo calcified lesions, so this detail of the registry should receive some emphasis.

Rather than focusing solely upon the efficacy of IVL, the authors somewhat uniquely report in detail on procedural complications including coronary dissections and perforations, hemodynamic instability requiring intervention, abrupt vessel closures, slow flow or no-reflow, and resuscitation. Composite major adverse cardiac events (defined as the composite of cardiac death, nonfatal target vessel myocardial infarction, or clinically driven target vessel revascularization) are also reported. Notably, the assessment of intraprocedural complications was done by a combination of investigator reporting as well as the use of an angiographic core laboratory.

The key message from this study is reassuring—procedural complications were relatively uncommon. Among 509 patients, 33 (6%) experienced procedural complications, and only 6 of these (1%) occurred immediately after IVL use. The most common complications were coronary dissections and hemodynamic instability (2% each), followed by coronary perforation (1%). Nearly all complications were successfully managed with standard interventions, and angiographic success, defined as residual stenosis <30%, was achieved in 97% of these cases. While complications were relatively rare, patients who experienced them had higher rates of major adverse cardiovascular events at 1 year (43% vs 9%), primarily driven by in-hospital events. This finding underscores an important point: even when complications are promptly treated, they still carry clinical implications extending beyond the procedure.

A notable strength of the study is its use of an angiographic core laboratory to assist in distinguishing between complications that occurred directly after IVL use and those occurring more generally during the PCI procedure (as a whole). This fits with conventional wisdom regarding the application of IVL-based acoustic energy during a low-pressure balloon inflation (in contrast to the potential for less-controlled vessel injury that occurs either during an atherectomy procedure or even the high-pressure inflation of a noncompliant balloon). That said, exonerating the IVL device based solely on the timing of when an angiographic complication occurred can be challenging, particularly given that application of a technology may indirectly influence the risk of a procedure’s subsequent steps. For example, the use of adjunctive devices such as rotational atherectomy prior to IVL represents a potential confounder when attempting to isolate IVL-specific complications. In this study, all 6 patients with complications reported immediately after IVL had undergone rotational atherectomy beforehand. These complications could, therefore, conceivably be attributed to either device (or both together). Additionally, among the group of patients treated with IVL experiencing complications, pre- and postdilation balloon sizes tended to be larger, although these differences were not statistically significant. Understanding the true risk profile of IVL requires considering the cumulative impact of the full PCI strategy rather than any single component of the overall procedure. In *toto*, these data support the hypothesis that many of the procedural complications in calcified lesions may not be specific to IVL itself, but instead reflective of the inherent risk posed by the lesion morphology and overall PCI complexity.

One of the most clinically relevant findings in this study was the identification of a higher balloon-to-artery ratio during predilation (prior to IVL) as an independent correlate of complications. This association suggests that more aggressive balloon sizing may increase the risk of vessel injury, particularly in heavily calcified arteries, and reinforces the importance of careful sizing and planning, ideally supported by intravascular imaging, to enhance procedural safety. Although intravascular imaging (intravascular ultrasound or optical coherence tomography) was used in over half of the cases, its application was not discernibly protocol-driven. Imaging plays a valuable role in optimizing device selection and ensuring adequate preparation before stent deployment. Given the observed relationship between balloon-to-artery ratio and complications, standardizing the use of imaging to carefully titrate lesion preparation could offer additional protection, particularly in anatomies with eccentric or diffuse calcification.

The overall rate of complications reported by the authors is in line with the REPLICA-EPIC18 registry, which reported a complication rate of 3.3% in a similar real-world population with acute coronary syndromes and complex anatomy.^7^ By contrast, earlier trials such as the Disrupt CAD series reported lower complication rates (0%-1.6%), likely reflecting stricter inclusion criteria and more structured treatment algorithms.^4^ Together, these data suggest that IVL remains a safe option in routine practice, although the risk profile may vary depending on case complexity and operator technique. Compared with other calcium modification tools such as rotational and orbital atherectomy, IVL continues to demonstrate a favorable safety profile. Trials such as ROTAXUS and ORBIT II reported similar rates of procedural success but higher rates of complications, particularly dissections and perforations.^8^^,^^9^ More recently, the large-scale ECLIPSE trial comparing orbital atherectomy with conventional balloon angioplasty in severely calcified lesions reported a core laboratory-assessed dissection rate of 6.9% and perforation in 1.8% of patients treated with orbital atherectomy; notably, these were no different from the use of noncompliant balloons alone.^10^ While the rates within the present study appear lower in comparison, as a retrospective, single-arm analysis with a limited total number of adverse events and inclusion of a significant proportion of patients with in-stent restenosis, cross-study comparisons are fraught with the challenges of dissimilar patient populations and core laboratories. Furthermore, operator decisions including the use of imaging, balloon sizing, and adjunctive tools were not standardized and may have introduced selection bias. In addition, some complication definitions within this study are less clearly specified than others.

Notwithstanding these limitations, the BENELUX-IVL registry reinforces the role of IVL as an effective calcium modification strategy in contemporary PCI. While complications were uncommon and generally manageable, their occurrence was associated with worse outcomes. A higher balloon-to-artery ratio appears to be a modifiable risk factor, reinforcing the utility of adjunctive intravascular imaging and careful procedural planning. As IVL adoption continues to expand, further prospective and directly comparative (and hopefully randomized) studies will be important to further refine best practices and optimize outcomes in patients with calcified coronary artery lesions.
